# The detection of a non-anemophilous plant species using airborne eDNA

**DOI:** 10.1371/journal.pone.0225262

**Published:** 2019-11-20

**Authors:** Mark D. Johnson, Robert D. Cox, Matthew A. Barnes

**Affiliations:** Department of Natural Resources Management, Texas Tech University, Lubbock, TX, United States of America; University of Hyogo, JAPAN

## Abstract

Genetic analysis of airborne plant material has historically focused (generally implicitly rather than as a stated goal) on pollen from anemophilous (wind-pollinated) species, such as in multiple studies examining the relationship of allergens to human health. Inspired by the recent influx of literature applying environmental DNA (eDNA) approaches to targeted-species and whole-ecosystem study, we conducted a proof-of-concept experiment to determine whether airborne samples reliably detect genetic material from non-anemophilous species that may not be releasing large plumes of pollen. We collected airborne eDNA using Big Spring Number Eight dust traps and quantified the amount of eDNA present for a flowering wind-pollinated genus (*Bouteloua*) and insect-pollinated honey mesquite (*Prosopis glandulosa*) that was not flowering at the time of the study. We were able to detect airborne eDNA from both species. Since honey mesquite is insect-pollinated and was not flowering during the time of this study, our results confirm that airborne eDNA consists of and can detect species through more than just pollen. Additionally, we were able to detect temporal patterns reflecting *Bouteloua* reproductive ecology and suggest that airborne honey mesquite eDNA responded to weather conditions during our study. These findings suggest a need for more study of the ecology of airborne eDNA to uncover its potential for single-species and whole-community research and management in terrestrial ecosystems.

## Introduction

Environmental DNA (eDNA) is shed genetic material that can be extracted from bulk environmental samples such as water, soil, or air [1,2]. eDNA analysis has been found to be typically faster, result in less disturbance to the environment, and be more sensitive than traditional fish, amphibian, and plant surveys [1,3,4]. While eDNA has been studied extensively in aquatic and terrestrial sediment samples, the rate of studies examining terrestrial plant DNA obtained from air samples has recently increased (e.g.,[5,6]).

Obtaining genetic information about terrestrial vegetation from airborne samples comes in part from a pioneering study by Longhi et al. [7] who developed a method to identify and quantify pollen grains through real-time polymerase chain reaction (PCR). Some of the first applications of this methodology discovered that genetic information could be obtained from pollen found within honey and collected by bees [8,9,10] and directly on insects for studies examining plant-pollinator interactions [11,12,13,14]. In addition to studying pollen in honey and plant-pollinator interactions, recent studies have also focused on plant material collected from air samples as opposed to off insects. These studies examining airborne eDNA have varied across many different fields and varying genetic complexities. For example, Folloni et al. [15] used real-time polymerase chain reaction (PCR) to examine how far pollen from genetically modified corn could be detected from source fields. Additionally, Mohanty et al. [16] studied the feasibility of using quantitative PCR to identify *Juniperus* pollen from Burkard spore traps and was able to detect the *Juniperus* pollen in a mixed pollen sample. While these studies examined airborne eDNA with PCR based methods, high throughput metabarcoding methods are becoming more and more common. For example, Kraaijeveld et al. [5] conducted a study that compared a metabarcoding survey for grass pollen allergens collected with Burkard spore traps to a traditional microscopic survey to help develop a new way to monitor pollen for pollen-allergic patients. Kraaijeveld et al. [5] found that the metabarcoding approach detected and identified grass pollen more efficiently than microscope-based techniques. Another metabarcoding study was completed by Leontidou et al. [6] with the goal of developing standardized protocols for the analysis of airborne pollen samples and specifically anemophilous taxa. Furthermore, Nuñez et al. [17] utilized next-generation sequencing to confirm it was possible to genetically identify bacterial, fungi, and pollen samples captured from a Hirst-type spore trap with the goal of understanding more about urban allergens and health hazards in the air. Lastly, in addition to outdoor samples, Korpelainen and Pietilainen [18] successfully characterized indoor pollen samples with a metabarcoding approach to understand the effects of pollen and indoor airborne particles on humans. While this brief review of airborne genetic sampling shows how various methods and questions can be addressed within these studies, it also highlights how these studies focus primarily on pollen detection.

As described above, previous studies have had an unstated assumption or biases that airborne eDNA samples exclusively represented pollen from wind-pollinated species rather than other materials and species with other pollination syndromes. However, some of the above studies suggested that there is a possibility for identifying plant fragments and material besides pollen [17,18], and Craine et al. [19] suggested there are other tissues and plant fragments besides pollen that contribute to settled indoor and outdoor dust. Therefore, we believe that these previous works provide a foundation for a more holistic view of airborne eDNA analysis. We believe that the definition and utility of airborne eDNA can be broadened to include more material shed from more sources. The goal of this paper is to expand the potential domain of airborne eDNA sampling beyond pollen from anemophilous species, increasing the relevance of the methods to diverse applications such as whole-community monitoring and detection of diverse endangered or invasive plant species.

A more complete understanding about what comprises the plant material collected as airborne eDNA will facilitate the growth of the field similarly to the recent expansion of aquatic and terrestrial sediment-based eDNA applications. For example, we know it is possible to detect pollen from wind-pollinated species with airborne eDNA sampling, but if airborne eDNA can detect other sources of plant DNA (e.g., flower and plant fragments) it may be possible to utilize airborne eDNA for whole-community monitoring instead of focusing only on wind-pollinated plants. We would be able to additionally detect both species that are not flowering and insect-pollinated species that would not be releasing large plumes of pollen into the air. It is known that plants desiccate and release fragments into the air, however no studies have attempted to use airborne eDNA to detect non-anemophilous plants. Therefore, our approach was to explore the capability of airborne eDNA to detect non-anemophilous species without the use of pollen by targeting species that would not be releasing large plums of pollen into the air (e.g., insect-pollinated species that were not flowering at the time).

We deployed Big Spring Number Eight (BSNE) dust traps on a short-grass rangeland to collect bulk environmental airborne samples. We designed and applied eDNA assays that targeted the *Bouteloua* genus which consists of wind-pollinated grama grasses that flowered during the time of study in the Poaceae family and honey mesquite (*Prosopis glandulosa)* which is an insect-pollinated tree species belonging to the Fabaceae family that was not flowering [20,21,22]. In this instance, *Bouteloua* acts as a positive control; since it is wind-pollinated, abundant, and flowering at the time of our study, if we failed to detect it, then a failure to detect Honey Mesquite would likely be due to our own methodological constraints (errors) rather than as a function of pollination syndrome. We believe this analysis serves as a proof-of-concept that represents a first step toward understanding how airborne eDNA can be expanded to fields such as whole plant community monitoring and is not limited to anemophilous species specific detection.

## Materials and methods

### Study site

We conducted research on the Texas Tech University Native Rangeland (33.60327 N, -101.9003 W), which serves as a teaching and research reserve for the University’s Department of Natural Resources Management. This site represents a short-grass rangeland, dominated by grasses and forbs, with sparse cacti and significant encroachment of honey mesquite. Additionally, the rangeland has the benefit of being isolated within an urban environment where there is limited *Bouteloua* and honey mesquite growth. This should help to limit the impact of eDNA from outside our study site for our specific species of interest. During the study, we measured precipitation amounts and timing. Wind-pollinated grasses of the *Bouteloua* genus, including *Bouteloua gracilis* and *Bouteloua dactyloides*, are common on the site and are known to flower and release pollen during the time of our study. The insect-pollinated honey mesquite (*Prosopis glandulosa*) is common on the site and was not flowering during our sampling period.

### Airborne eDNA survey

**Fig 1:**
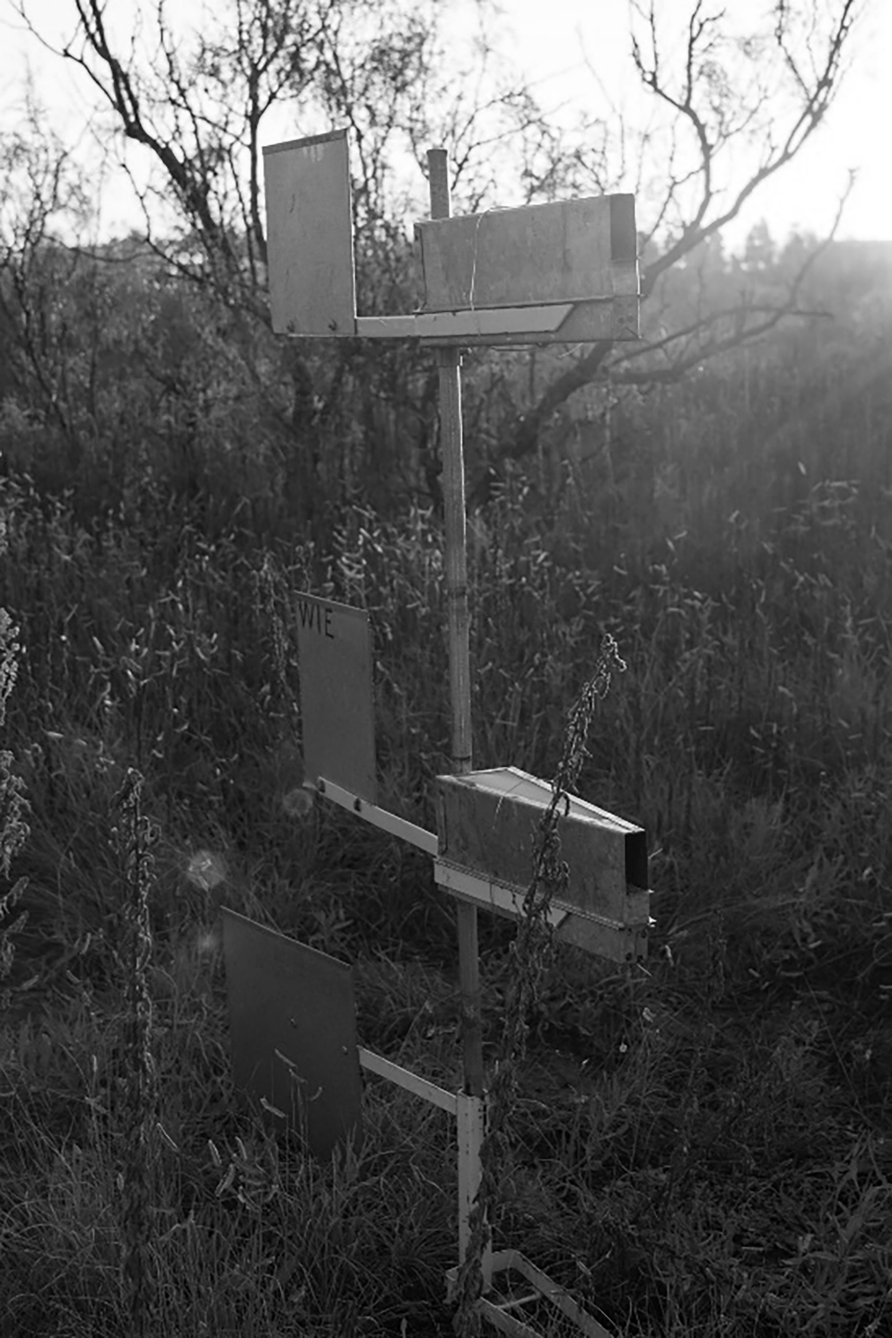
Study site. The 130 acre (33.60327 N, -101.9003 W) Texas Tech University Native Rangeland study site and the locations of our nine Big Spring Number Eight dust traps on the landscape.

**Fig 2:**
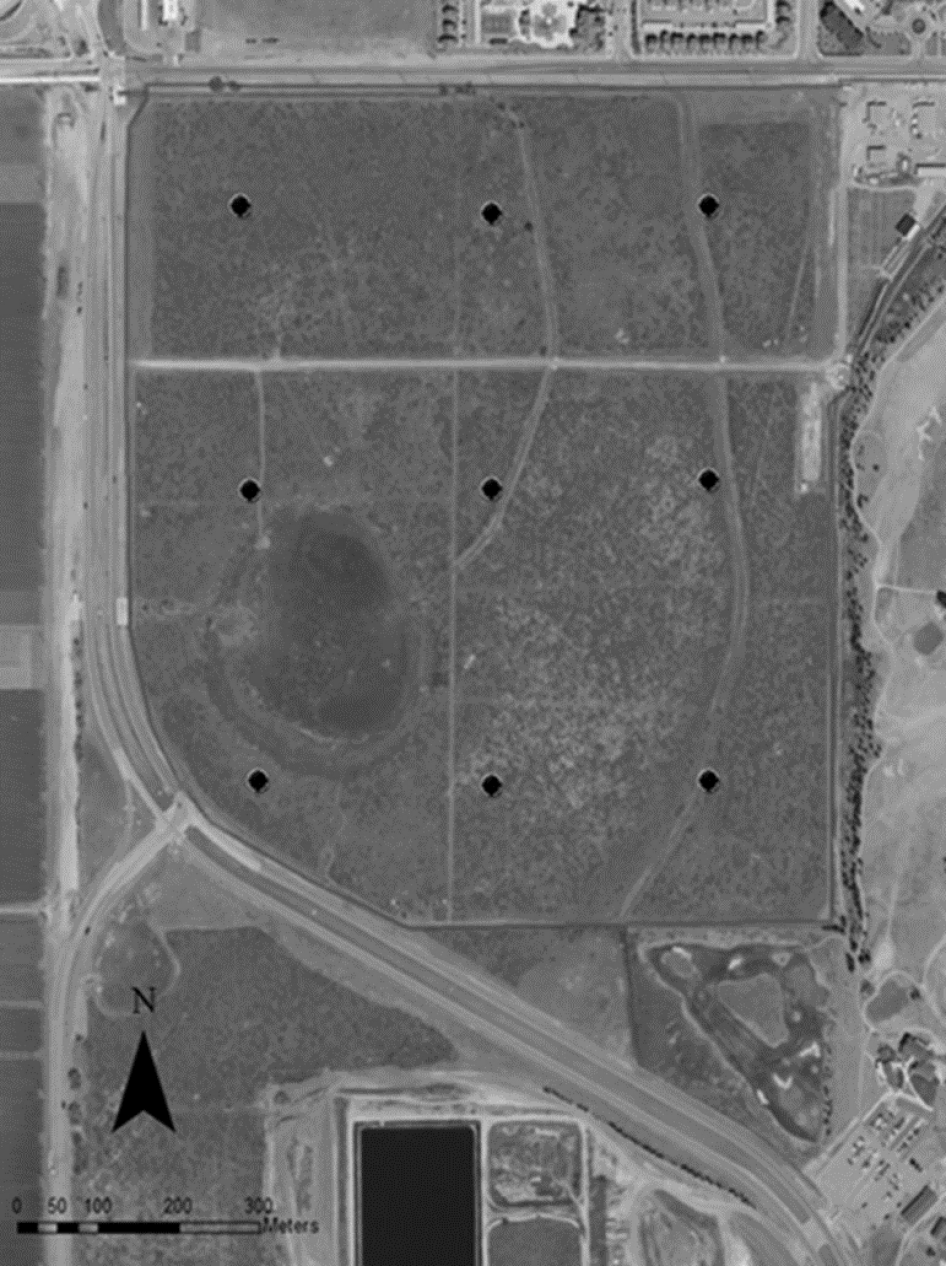
The BSNE dust traps that collected airborne eDNA throughout this study.

To collect airborne eDNA, we deployed nine Big Spring Number Eight (BSNE) dust traps in a grid across the Rangeland (Fig 1). BSNE traps are commonly used for long- and short-term dust research [23]. Our traps were designed to collect >10μm dust particles, which is also suitable to collect eDNA from airborne samples [24, 25]. The BSNE traps consisted of two triangular traps 0.914 m and 0.406 m above the ground. The traps had an opening vertically in the front and a vent on top, which allowed air to move through the trap, depositing any material into a collection tray below (Fig 2). The nine traps were deployed on July 24, 2016 and sampled every two weeks until October 2, resulting in five total sampling events. At each sampling event, each trap was rinsed with approximately 1L deionized water and the water was collected into individual, sterile 1L-bottles. BSNE traps consist of two collection trays, so to avoid issues of pseudoreplication [26] and collect as much bulk material as possible, each collection tray was washed with approximately 0.5L deionized water and combined into a single sample. Water samples were transported to the laboratory within a cooler and vacuum filtered with 3μm Isopore membrane filters (MilliporeSigma). Filters were then stored at -20°C until DNA extraction.

### Laboratory analysis

Total genomic DNA was extracted from each filter using a DNeasy Plant Mini Kit (Qiagen, Hilden, Germany) following manufacturer’s protocols except that the approximately 47mm diameter filters were folded and ground with a sterile plastic pestle within a collection tube for approximately 15 seconds. This was found to increase DNA yields in pilot trials, presumably by increasing agitation of eDNA material from the filter and increasing exposure to extraction reagents. We also took precautions to ensure there was no contamination, including the incorporation of extraction blanks (i.e., clean filters treated as “real” samples with each batch of DNA extractions), bleaching of laboratory surfaces, use of sterile gloves, and physical separation between labs where DNA extraction and qPCR were performed. Once the extractions were completed for each sample (containing 200 μl of DNA), they were stored at -20° C to await qPCR. We had originally conducted a pilot experiment to examine how best to preform extractions and to test qPCR protocol and found that our airborne eDNA samples were greatly impacted by PCR inhibition. This inhibition resulted in complete inhibition of our PCR products in most cases (no amplification) and the samples that did amplify had artificially low quantities. To control for this, all samples were diluted tenfold with distilled water.

We completed qPCR reactions on a QuantStudio 3 Real-Time qPCR machine (ThermoFisher Scientific, Foster City, California). For the detection of the *Bouteloua* genus, each 25 μl qPCR reaction contained 1x PowerSYBR Green qPCR Master Mix, 1μM forward and reverse primer (5′‐ACCCGTTCCTGGAGAAGATAGT‐3′ 5′‐CAGGAGGAATTCGTAGATCCTCCA‐3′; S1 File), and 2μl 1:10 diluted DNA extract. The honey mesquite qPCR reaction likewise was 25 μl and contained 1x PowerSYBR Green qPCR Master Mix, 1μM forward and reverse primer (5′‐CTGAAGAAGCAGGTGCTGCG‐3′ 5′‐TTGAGTTTCTTCTCCAGGAACAGG‐3′; S1 File), and 2μl 1:10 diluted DNA extract. The thermocycling program for the *Bouteloua* genus used an initial 95°C step for ten minutes followed by two steps: 15 seconds at 95° C and 1 minute at 66° C for 40 cycles and a final melt curve analysis. The thermocycling program for the honey mesquite primer used an initial 95°C step for ten minutes followed by two steps: 15 seconds at 95° C and 1 minute at 70.1° C for 40 cycles and a final melt curve analysis. For the Bouteloua assay, samples were run in triplicate, and for the honey mesquite assay, the samples were run in quintuplicate. Since *Bouteloua* in this instance acts as a positive control in our study, we ran the samples in triplicate. For honey mesquite, which is suspected to be much rarer and the experimental target of our sampling, samples were run in quintuplicate. Target eDNA concentration in each reaction was quantified using a five-point standard curve standard curve based on 1:10 serial dilutions of tissue-derived DNA from blue grama (*Bouteloua gracilis*; the most common *Bouteloua* genus species on the study site) or honey mesquite. These dilutions for the *Bouteloua* and honey mesquite ranged from 5340 pg/μl to 0.53 pg/μl and 1060 pg/μl to .106 ng/μl, respectively. Data were recorded as both presence/absence as well as average target eDNA concentration per site. The data described below comes from eDNA that exists in very low concentrations which oftentimes results in replicates genuinely not containing target DNA [27]. To adjust for this we assigned the replicates and sites that recorded non-amplifications a value of 0 following the recommendation of Ellison et al. [27] for samples that are expected to be low in concentration and include stochastic non-detections. All reactions included no-template controls to ensure no contamination occurred.

## Results

**Fig 3:**
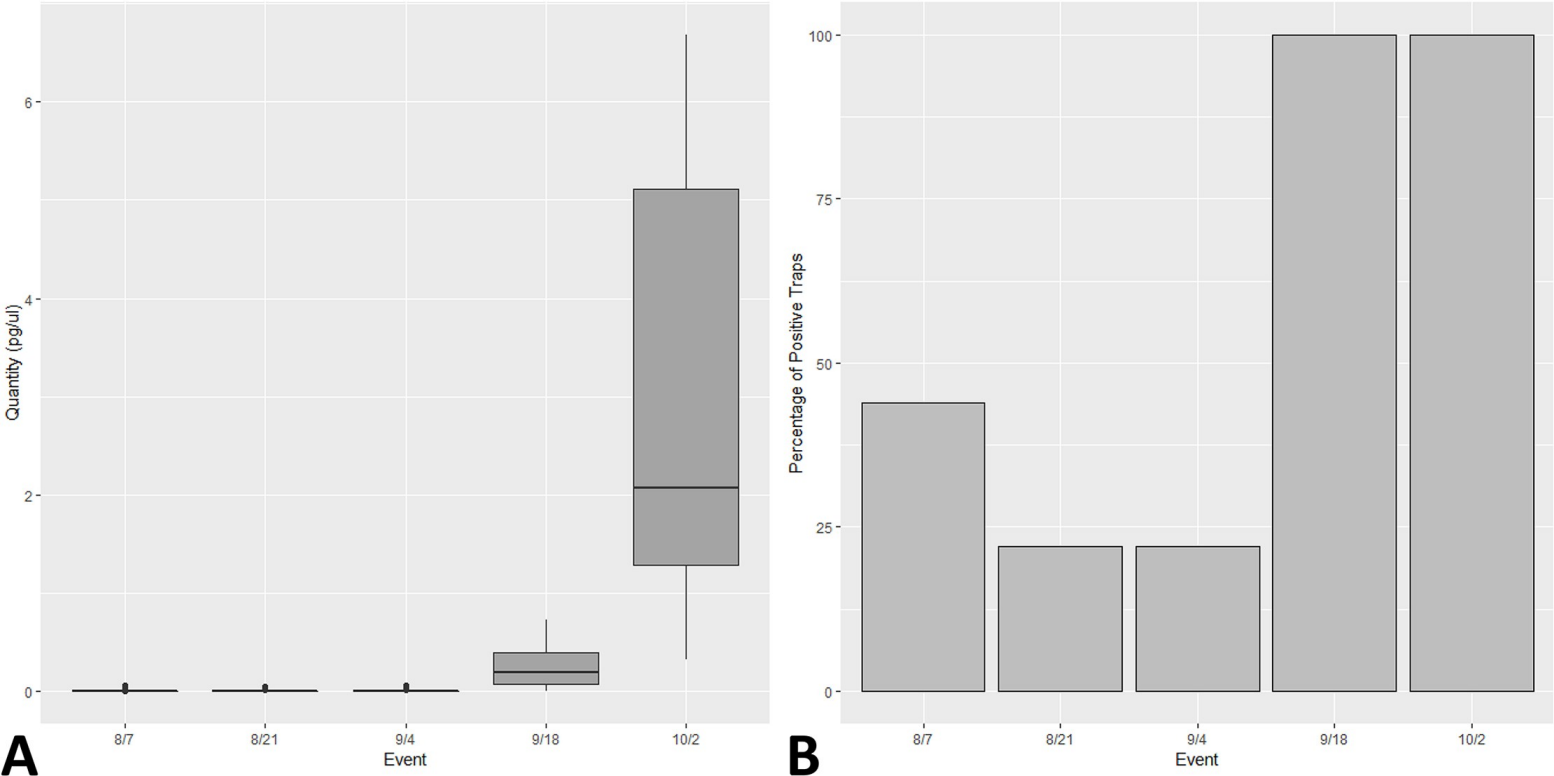
*Bouteloua* eDNA increased over time in correspondence with pollination events. (A) The quantity of *Bouteloua* eDNA over the course of five sampling events across all nine BSNE traps. Boxes extend to the upper and lower quartile range while showing the median as dark solid line. Whiskers extend to the 95% confidence interval of the data and outliers are represented by circles. (B) The percentage of BSNE dust traps that detected a significant amount of *Bouteloua* airborne eDNA. The *Bouteloua* genus eDNA was detected 44, 22, 22, 100, and 100 percent of the time for events 1,2,3,4, and 5 respectively.

**Fig 4:**
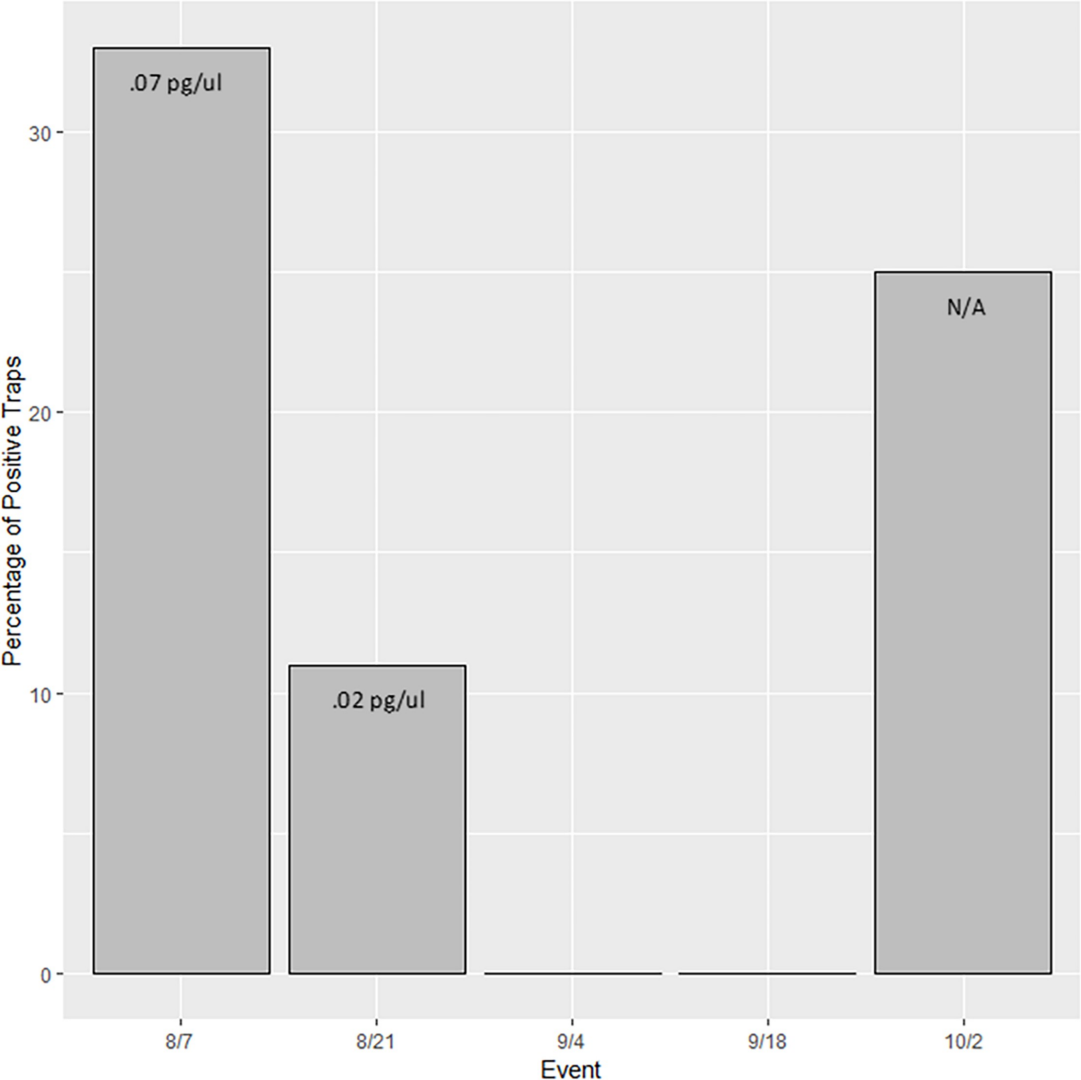
Honey mesquite eDNA was detected during our sampling events. The percentage of traps that detected honey mesquite airborne eDNA across five sampling events with the quantity of each event on top of each bar. The percentage of positive honey mesquite samples was 33, 11, 0, 0, and 25 percent during sampling events 1, 2, 3, 4, 5 respectively. Notably, there is no eDNA amount for the detections associated with event 5 because these detections were found during a PCR with no standard curve.

To increase our understanding of what comprises airborne eDNA, we attempted to detect flowering wind-pollinated *Bouteloua* genus plants and non-flowering insect-pollinated honey mesquite using passive dust samplers (S2 File). No amplification occurred in any extraction blank or no-template qPCR control throughout the experiment. eDNA from the *Bouteloua* genus was detected during every sampling event from July 24 to October 2 and at all trap locations. The percent of positive detections for the *Bouteloua* genus did vary over time (Fig 3A). Additionally, the average concentration of *Bouteloua* eDNA across all nine traps was low for the first three sampling events and then demonstrated a large increase during events 4 and 5 (Fig 3B). The concentration of *Bouteloua* eDNA went from 0.005 pg/ul in the first two sampling events and 0.004 pg/ul in the third to 0.256 pg/ul in the fourth event and lastly, 3.05 pg/ul for the final sampling event. We detected eDNA from honey mesquite during three of the five sampling events, although detection varied across trap locations. The percent of positive detections for honey mesquite also varied over time (Fig 4). The concentration of honey mesquite was low throughout the sampling events in contrast to the relatively high concentrations observed for *Bouteloua* eDNA (Fig 4).

**Fig 5:**
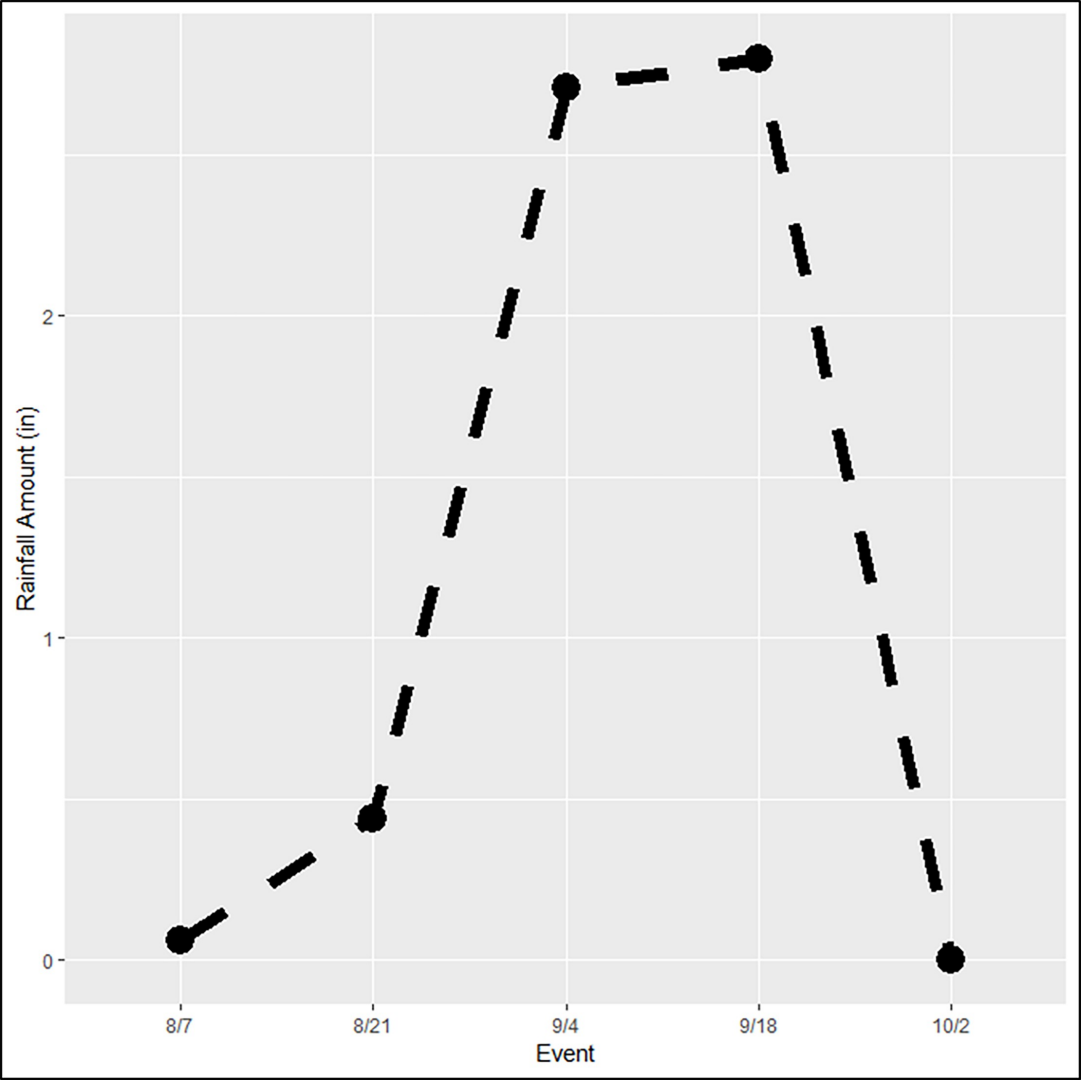
The amount of rainfall that was recorded throughout the sampling period in inches.

During our experiment, rainfall was measured and obtained from the National Centers for Environmental Information (NOAA). Minimal precipitation occurred during the period encompassing events 1 and 2 (Fig 5). However, rainfall increased dramatically in the two weeks leading up sampling events 3 and 4 before it fell again for the final event.

## Discussion

Our results confirm that airborne eDNA comes from a variety of sources and that we can detect insect-pollinated (and thus non-anemophilous) non-flowering species. Although research on airborne genetic material from terrestrial plants has often implicitly focused on pollen from anemophilous species and its relationship to human health [5,18], our work helps to demonstrate a broader applicability of such methods to more species. With this foundation, the field of airborne eDNA analysis can be expanded into new and exciting research and management applications, such as monitoring diverse plant species and plant communities, detecting invasive species, and protecting threatened or endangered species.

Notably, we found that while both species could be detected, there were species-specific differences in the concentrations and regularity of the eDNA detected. For example, the *Bouteloua* genus airborne eDNA concentration increased sharply over the course of our study. We attribute this trend to pollination syndrome, seasonality, and the ecology of blue grama, *Bouteloua gracilis*, the most common *Bouteloua* species on our study site. Blue grama goes dormant during the summer and then flowers and releases pollen in early fall [28, 29], which aligns with the large increase in *Bouteloua* detection and quantity we observed for events 4 and 5 toward the end of September as shown in Fig 3A and 3B. This observation suggests that airborne eDNA methods can detect the plume of eDNA from flowering wind-pollinated species during their pollination season when the amount of eDNA is maximized in the air while also being able to detect the species during less prolific pollination times. Similar results have been observed in previous pollen studies [5]. Future studies should further explore the generality of the relationship between airborne eDNA dynamics and species ecology. In other words, do we see the different stages within species ecology (growth, senescence, dormancy, and pollen release) reflected in airborne eDNA detection and how universal are these patterns. If such a relationship is common, it will prove useful for informing the timing of airborne eDNA plant community surveys.

Honey mesquite airborne eDNA, while detectable, was present in lower concentrations and less consistently detected overall. Since honey mesquite is insect-pollinated and flowers in the spring [21,30] this low concentration of eDNA could be the result of loss of material over time due to wind and disturbance. While it is possible that pollen was brought to the traps from insects, this would seem to be unlikely since the trees were not flowering at this time, thus producing no pollen for the insects. However, as the trees lose their leaves or their leaves get fragmented, the DNA within these plant parts could be detected by wind traps. Indeed, during sampling we observed several vegetative fragments within the samples which help to provide evidence of this material release. As mentioned above, being non-flowering at the time and an insect-pollinated species, this time this time of year should be one the most challenging times to collect honey mesquite eDNA, yet we were still able to detect this species in airborne eDNA samples.

We did not detect honey mesquite in any of our nine traps during the third and fourth sampling events (Fig 4). One explanation for this change in detection rate could relate to an interaction between the ecology of honey mesquite airborne eDNA and local weather. The two weeks leading up to the fourth and fifth sampling events experienced large amounts of rain which could have impacted our ability to collect and detect the honey mesquite eDNA (Fig 5). This may suggest that the honey mesquite airborne eDNA is less likely to travel horizontally across the landscape during precipitation events since the rain is essentially forcing the eDNA down toward the ground out of the air. On the other hand, we were still able to detect *Bouteloua* eDNA during this time period since *Bouteloua* airborne eDNA was considerably more abundant in the environment. As a result, it would appear that the *Bouteloua* eDNA had the opportunity to make it into the BSNE traps after the rains whereas the honey mesquite airborne eDNA, which is in much lower concentrations, was not detected. Future studies should continue to explore how airborne eDNA responds to environmental conditions, specifically considering how precipitation may impact airborne eDNA detection success. Finally, we found that the amount of airborne eDNA during a single sampling event was not consistent across all nine BSNE traps for either of our target species (Fig 3). This suggests that there were some factors that differed between trap locations that could have influenced eDNA collections. Some options of what these factors entail could be related to the biomass of each specific plant around each trap, wind conditions at each trap, or how airborne eDNA travels in the air column, among others.

By showing that airborne eDNA is not limited solely to the detection of pollen from wind-pollinated flowering species, we expand the use of airborne eDNA past pollen detection and human health applications. However, before we are able to meet this ambitious goal there is much more that needs to be understood about airborne eDNA. In these final paragraphs, we would like to highlight both future research directions that we feel would benefit airborne eDNA studies the most and potential applications. First, we must understand more about the ecology of airborne eDNA. The ecology of airborne eDNA refers to its origin, state, transport, and fate, and how these aspects interact with the biotic and abiotic environment. For instance, we collected samples every two weeks, but there is a possibility this could be either too long or too short resulting in varying amounts of eDNA collected. Furthermore, there are multiple types of passive traps that may be able to detect airborne eDNA more efficiently than others such as marble dust traps and Modified Wilson and Cooke traps [31]. Finally, understanding how far airborne eDNA can move through the air and be detectable from common and rare plants needs to be explored further.

With the use of both pollen and plant fragments from insect-pollinated and non-flowering plants, one of the many applications of airborne eDNA could be for the rapid detection of invasive species. Rapid detection and response represent keystones of successful invasive species management and could potentially be achieved through airborne environmental DNA [32]. Airborne eDNA can also be used to protect and learn more about endangered species. In aquatic systems, eDNA has already been considered and utilized to monitor and find endangered species [33,34]. Additionally, airborne DNA has the potential to be used in conjunction with traditional pollen identification methods (microscopy) to help preform more accurate whole plant community surveys that target species not only releasing pollen into the air. Lastly, next-generation metabarcoding techniques could be applied to airborne eDNA surveys and represent an even more efficient sampling method for plant communities. With its many potential applications, airborne eDNA represents an exciting new addition to the plant community analysis toolbox.

## Supporting information

S1 File Methods for primer design. (PDF)

S2 File qPCR data for both the *Bouteloua* genus and honey mesquite quantities. (XLSX)
