# Supplementary material for: The detection of a non-anemophilous plant species using airborne eDNA
Source: PLoS One. 2019 Nov 20;14(11):e0225262. doi: 10.1371/journal.pone.0225262 (PMC6867632; doi:10.1371/journal.pone.0225262)
Supplement: S1 File — (PDF) [file pone.0225262.s001.pdf]

S1 Appendix: Methods for primer design

We selected the *Bouteloua* genus (wind-pollinated blue grama and buffalo grass) and *Prosopis glandulosa* (insect-pollinated honey mesquite) to test for the effect of pollination syndrome on the detection airborne eDNA. To create primers that could detect this genus and species, we first gathered all the available ribulose-bisphosphate carboxylase (rbcL) chloroplast sequences for our target organisms from NCBI GenBank (Clark, Karsch-Mizrachi, Lipman, Ostell, Sayers 2016). We also gathered any rbcL sequences available from species known to be on our study site from NCBI GenBank (Table 1). The accessed non-target sequences served as targets to avoid during primer design. Next, all of the sequences were aligned using BioEdit Sequence Alignment Editor (Hall 1999). Once aligned, we used DECIPHER's Design Primers web tool (http://decipher.c ee.wisc.edu/DesignPrimers.html; Wright 2014) to produce candidate primers for both the *Bouteloua* genus and honey mesquite. After we obtained the candidate primers, we assessed their ability to amplify genomic DNA extracted from 90 mg field-collected plant tissue for their respective genera and species. We selected the primer that amplified the target DNA the most often and whose PCR amplification curves were the most consistent. Additionally, to ensure specificity, the primer set that amplified our target DNA the best was also tested against the most common species on our study site, which were identified through a line-point intercept and visual plant survey in the summer of 2016 (*Bouteloua gracilis*, *Prosopis glandulosa*, *Laennecia coulteri*, *Helenium amarum var. badium*, *Solanum elaeagnifolium*, *Cucurbita foetidissima*, *Nassella tenuissima*, *Bouteloua dactyloides*, and *Helianthus ciliaris*). Once we ensured that our primers could successfully amplify the target genus or species and did not amplify any of the most common species on our study site we made our selection (Table 2).

References

1. Clark K. Karsch-Mizrachi I., Lipman D., Ostell J., Sayers E. (2016). GenBank. Nucleic Acids Research, 44(Database Issue), D67-D72.

2. Hall T. (1999). BioEdit: a user-friendly biological sequence alignment editor and analysis program for Windows 95/98/NT. Nucleic Acids Symposium Series, 41, 95-98.

3. Wright E. (2014). Exploiting extension bias in PCR to improve primer specificity in ensembles of nearly identical DNA templates. Environmental Microbiology, 16(5). doi:10.1111/1462-2920.122259.

Tables

Table 1. The species found within the Texas Tech University Native Rangeland. The species in bold had sequences for the rbcL region of interest available in NCBI GenBank.

Ailanthus altissima	***Cucurbita foetidissima***	***Krameria lanceolata***	*Quincula lobata*
Ambrosia psilostachya	***Cylindropuntia leptocaulis***	***Lactuca serriola***	*Ratibida columnifera*
Ammoselinum popei	***Cynodon dactylon***	*Laennecia coulteri*	*Ratibida tagetes*
Amphiachyris dracunculoides	*Cyperus uniflorus*	***Lamium amplexicaule***	*Rumex hymenosepalus*
Aristida adscenscionis	*Dalea nana*	***Lepidium densiflorum***	*Salsola tragus*
Aristida purpurea	***Daucus pusillus***	***Lithospermum incisum***	*Salvia reflexa*
Artemisia filifolia	*Delphinium carolinianum*	*Lygodesmia texana*	*Schizachyrum scoparium*
Berlandiera lyrata	***Descurainia pinnata***	*Machaeranthera tanacetifolia*	*Senecio flaccidus*
Bothriochloa barbinodis	*Digitaria californica*	***Malva neglecta***	*Senna pumilio*
Bothriochloa saccharoides	*Digitaria cognata*	*Malvella lepidota*	*Setaria leucopila*
Bouteloua curtipendula	*Ditaxis humilis*	***Marrubium Vulgare***	*Solanum elaegnaifolium*
Bouteloua dactyloides	*Echinocactus texensis*	*Mimosa aculeaticarpa*	*Solanum rostratum*
Bouteloua gracilis	***Echinochloa crus-galli***	*Mirabilis linearis*	***Sonchus asper***
Brickellia eupatorioides	***Elymus elymoides***	***Monarda citriodora***	*Sophora nuttalliana*
Bromus catharticus	***Engelmannia peristenia***	*Muhlenbergia arenicola*	***Sorghum halepense***
Cenchrus incertus	*Eragrostis cuptipedicelata*	***Muhlenbergia paniculata***	*Sphaeralcea angustifolia*
Centaurea melatonsis	***Eragrostis curvula***	*Muhlenbergia torreyi*	***Sphaeralcea coccinea***
Chaetopappa ericoides	*Erigeron modestus*	*Nama hispidum*	***Sporobolus cryptandrus***
Chamaesaracha sordida	*Euphorbia lata*	***Nassella tenuissima***	***Suckleya suckleyana***
Chenopodium album	*Euphorbia spathulata*	*Oenothera canescens*	***Taraxacum officinale***
Chenopodium leptophyllum	***Evolvulus nuttallianus***	*Oenothera macrocarpa*	*Teucrium laciniatum*
Chinopodium incanum	***Glandularia bipinnatifida***	***Oenothera suffrutescens***	*Tridens albescens*
Chloris cucullata	*Grindelia ciliata*	*Opuntia phaeacantha*	***Ulmus pumila***
Chloris verticillata	***Grindelia squarrosa***	***Oxalis dillenii***	***Verbena bracteata***
Cirsium ochrocentrum	***Gutierrezia sarothrae***	*Oxalis priceae*	*Verbesina encelioides*
Cirsium undulatum	***Helenium amarum***	*Persicaria pensylvanica*	*Vernonia marginata*
Cirsium vulgare	***Helianthus annuus***	*Picradeniopsis woodhousei*	***Xanthisma spinulosum***
Commelina erecta	*Helianthus ciliaris*	***Plectocephalus americanus***	*Xanthisma texanum*
Convolvulus arvensis	*Heterotheca villosa var. nana*	*Portulaca oleracea*	***Xanthium strumarium***
Conyza canadensis	*Hopia obtusa*	*Portulaca pilosa*	***Yucca glauca***
Coreopsis tinctoria	***Hordeum jubatum***	*Proboscidea louisianica*	*Zinnia grandiflora*
Croton texensis	*Hymenopappus tenuifolius*	***Prosopis glandulosa***	*Ziziphus obtusifolia*
Cryptantha minima	***Kochia scoparia***	*Psoralidium tenuiflorum*	

Table 2. The *Boutleoua* genus primer and honey mesquite primer selected for use within this experiment	
Target	Sequence
Bouteloua genus	Forward- 5’- ACCCGTTCCTGGAGAAGATAGT- 3’<br>Reverse- 5’- CAGGAGGAATTCGTAGATCCTCCA- 3’
Honey Mesquite	Forward- 5’- CTGAAGAAGCAGGTGCTGCG-3’<br>Reverse- 5’-TTGAGTTTCTTCTCCAGGAACAGG-3’
